# Egg Consumption in Infants is Associated with Longer Recumbent Length and Greater Intake of Several Nutrients Essential in Growth and Development

**DOI:** 10.3390/nu10060719

**Published:** 2018-06-04

**Authors:** Yanni Papanikolaou, Victor L. Fulgoni

**Affiliations:** 1 Nutritional Strategies, 59 Marriott Place, Paris, ON N3L 0A3, Canada; 2 Nutrition Impact, 9725 D Drive North, Battle Creek, MI 49014, USA; vic3rd@aol.com

**Keywords:** NHANES, infants, nutrients, eggs, growth

## Abstract

Nutrient intake during infancy is critical for healthy growth and development. The present study examined egg consumption and associations with nutrient intakes, markers of growth and weight-related measures in infants 6–24 months of age (*N* = 561) compared to infant egg non-consumers (*N* = 2129). Egg consumers were defined as those infants consuming eggs (i.e., with the exclusion of mixed dishes) during a 24-h dietary recall. Associations with nutrient intakes and markers of growth variables were evaluated using data from What We Eat in America, the dietary component of the National Health and Nutrition Examination Survey, 2001–2012. Mean energy and nutrient intakes were adjusted for the sample design using appropriate survey parameters and sample weights. Egg consumption was associated with greater energy intake compared to infants not consuming eggs (1265 ± 27 vs. 1190 ± 14 kcal/day; *p* = 0.01). Infant consumers of eggs also had greater protein (48 ± 0.7 vs. 41 ± 0.4 g/day), total choline (281 ± 6 vs. 163 ± 2 mg/day), lutein + zeaxanthin (788 ± 64 vs. 533 ± 23 mcg/day), α-linolenic acid (0.87 ± 0.02 vs. 0.82 ± 0.01 g/day), docosahexaenoic acid (DHA) (0.04 ± 0.02 vs. 0.02 ± 0.001 g/day), vitamin B12 (4.2 ± 0.1 vs. 3.7 ± 0.1 mcg/day), phosphorus (977 ± 15 vs. 903 ± 8 mg/day), and selenium (67 ± 1 vs. 52 ± 0.6 mcg/day; all *p*-values < 0.05). Egg consumers also had greater consumption of total fat (50 ± 0.7 vs. 45 ± 0.3 g/day), monounsaturated fat (17 ± 0.3 vs. 15 ± 0.1 g/day), saturated fat (20 ± 0.4 vs. 18 ± 0.2 g/day), and sodium (1663 ± 36 vs. 1418 ± 19 mg/day), with lower added sugar (4.7 ± 0.3 vs. 6.1 ± 0.2 tsp eq/day), and total sugar (87 ± 2 vs. 99 ± 1 g/day; all *p*-values < 0.05) vs. non-consumers of eggs. Egg consumption was also associated with lower intake of dietary folate, iron, magnesium and niacin relative to non-consumers of eggs. Egg consumption in infants was associated with longer recumbent length when compared to non-consumers of eggs (79.2 ± 0.2 vs. 78.7 ± 0.1 cm; *p* = 0.03). No associations were observed when comparing body weight. When compared to non-consumers of eggs and regardless of food security, poverty-income-ratio and Women, Infants and Children (WIC) supplemental nutrition status, egg consumption was associated with greater lutein + zeaxanthin intake per day. The current analyzes show that consumption of eggs in infant 6–24 months of age is linked with several nutrient intakes, including higher protein, lutein + zeaxanthin, choline, B12, selenium and phosphorus; and lower added and total sugars relative to non-consumers. Egg consumers also have less of several nutrients to be encouraged and a higher intake of nutrients to limit, thus presenting opportunities for educational strategies to potentially increase consumption of nutrient-dense foods in combination with eggs.

## 1. Introduction

While, historically, eggs have been discouraged from inclusion in a healthy maternal and lactating dietary pattern, key recommendations from the current 2015–2020 Dietary Guidelines for Americans (2015–2020 DGA) policy report includes vegetables, fruits, grains, dairy products, and a variety of protein foods, including eggs, seafood, lean meats, legumes, nuts and soy products. The 2015–2020 DGA states that eggs are nutrient-dense foods when prepared with little or no added solid fats, sugars, or sodium [1]. Similarly, the previous introduction of solid food recommendations for infants supported the avoidance of early introduction of eggs and egg-containing foods for infant dietary patterns due to concerns related to atopic disease. In 2003, a collaborative effort between the Nutritional Committees from the American Academy of Pediatrics (AAP), the European Society for Pediatric Allergology and Clinical Immunology, and the European Society for Pediatric Gastroenterology, Hepatology, and Nutrition published recommendations for delaying the introduction of eggs until two-years of age to prevent food allergy in children [2], with the American College of Allergy, Asthma and Immunology presenting similar guidelines in 2006 [3]. The current review of scientific evidence by AAP has established a lack of convincing evidence in support of delaying the introduction of eggs. Therefore, some health professionals believe the introduction of eggs in infancy, after four to six months of age, does not increase the risk of allergy or egg-related sensitivity [4,5].

High-quality protein foods, like eggs, can play an important role in healthy growth and development in infants, children and adolescents [6]. For about 70 kilocalories, one large 50 g egg contributes a large range of essential vitamins and minerals and bioactive compounds [7]. Eggs are also considered a source of high-quality protein, with an amino acid profile against which other protein sources are compared in experimentation and scientific claims [6]. Additionally, eggs are natural sources of high concentrations of choline, an essential nutrient identified by the National Academy of Medicine (formerly the Institute of Medicine) within the last two decades [8]. One large egg provides approximately 147 mg of total choline and represents one of the richest sources of dietary choline in the diet [7,9]. While several biological pathways regulate growth and development in infants and toddlers, choline is a key nutrient involved in numerous biological reactions. In fact, choline is a nutrient that plays an integral role in the structural integrity of cell membranes, methyl metabolism, cholinergic neurotransmission, transmembrane signaling, lipid and cholesterol transport, and metabolism [6,7,8,9]. Previous evidence has established the importance of choline availability in normal development and function of the infant brain [6,8]. Additionally, one 50 g egg provides approximately 30 mg docosahexaenoic acid (DHA) [7], which translates to 30% of the recommendation from the European Food Safety Authority for children >6 to 24 months of age (100 mg DHA/day) [10]. The role of DHA in infant growth and neurodevelopment and its functional attributes have been extensively documented [11].

Previous randomized controlled trials have demonstrated a benefit of egg consumption in infants. Indeed, one egg per day for six-months versus control (no eggs) in infants 6 to 9 months of age, led to increased length-for-age (i.e., linear growth) and weight-for-age z scores, in addition to a reduced prevalence of stunting and underweight [12]. In earlier work, researchers showed that one egg per day significantly increased choline and DHA intake relative to a control group [13]. Collectively, these data suggest that early introduction of eggs may significantly impact growth-related outcomes in addition to brain function and development, while also helping to contribute to daily nutrient intakes.

At present, the 2015–2020 DGA food patterns provides guidance on the amounts of foods to consume from the food groups (fruits, vegetables, grains, protein foods, and dairy) and their respective food sub-groups (dark green vegetables, orange and red vegetables, starchy and other vegetables, beans and peas, whole grains, enriched grains, meat, poultry and eggs, nuts, seeds, soy products and seafood), all of which are based on nutrient-dense foods [1]. Currently, the Dietary Guidelines for Americans has provided nutrition and food-based recommendations for individuals aged two years and older, with no comprehensive dietary guidance for infants or toddlers less than two-years-old. In 2012–2013, the United States Department of Agriculture, in collaboration with Health and Human Services, partnered with National Institutes of Health to explore and identify research gaps, data needs and systematic reviews with the goal of creating dietary recommendations for children from birth to 24 months of age, which would be included as part of the 2020–2025 Dietary Guidelines for Americans (2020–2025 DGA) [14].

While the process is underway for establishing dietary guidance for infants as part of the 2020–2025 DGA, at present, there are no studies that have examined nutrient intakes, diet quality and growth marker associations between infant egg consumers and non-consumers. Therefore, the aim of the present analysis was to examine egg consumption and associations with nutrient intakes, markers of growth and weight-related measures in infants 6–24 months of age and make comparisons to non-consumers of eggs.

## 2. Experimental Section

The National Health and Nutrition Examination Survey (NHANES) is a nationally-representative, cross-sectional survey of U.S. non-institutionalized, civilian residents. NHANES data are collected by the National Center for Health Statistics of the Centers for Disease Control and Prevention. Written informed consent was obtained for all participants or proxies, and the survey protocol was approved by the Research Ethics Review Board at the National Center for Health Statistics. Data from six NHANES datasets (2001–2002; 2003–2004; 2005–2006; 2007–2008; 2009–2010; and 2011–2012) were combined for the present analyzes in infants from 6 to 24 months of age [15]. Nutrient intake data for NHANES are from the relevant United States Department of Agriculture (USDA) Food and Nutrient Database for Dietary Studies (FNDDS) versions 5.0 and 6.0 for NHANES 2009–2010 and 2011–2012, respectively [16,17]. FNDDS are databases that provide the nutrient values for foods and beverages reported in What We Eat in America (WWEIA) [18], the dietary intake component of NHANES for each data release. The WWEIA Food Categories provide an application to analyze food and beverages as consumed in the American diet. The classification scheme includes over 150 unique categories, and there are 15 main food groups and 46 subcategories of foods.

WWEIA is collected using the Automated Multiple Pass Method (AMPM). USDA’s AMPM represents a validated dietary data collection instrument that provides an evidence-based, efficient and accurate format for collecting dietary intake data for large-scale national surveys [19]. The AMPM protocol is updated for each 2-year collection of WWEIA to account for the evolving food supply and address any research needs. AMPM is a fully computerized recall method that uses a 5-step interview: (1) quick list; (2) forgotten foods; (3) time and occasion; (4) detail cycle; and (5) final probe. AMPM includes an extensive compilation of standardized food-specific questions and possible options [19]. Dietary recall status codes are used by interviewers in both the Individual Foods and Total Nutrient Intakes files to indicate the quality and completeness (i.e., reliable or unreliable) of a survey participant’s response to the dietary recall section [19,20]. Further research has also demonstrated the accuracy of the AMPM by comparing reported energy intake to total energy expenditure using the doubly labeled water technique [21].

### 2.1. Subjects

In the present analyzes, the combined NHANES dataset sample included male and female infant participants classified as either egg consumers (*N* = 561) or non-consumers (*N* = 2129), that ranged in age from birth to 24 months, who had reliable and complete 24 h dietary recall interview data from WWEIA. Egg consumers were defined as those infants consuming eggs (i.e., with the exclusion of mixed dishes) during the 24 h dietary recall. Egg intake was determined using food codes in WWEIA category number 2502 ‘Eggs and omelets’ with exclusions for FNDDS group number 33 ‘Egg’ substitutes and FNDDS group number 312 ‘Other poultry eggs’.

Trained individuals completed the 24 h dietary recalls using USDA’s AMPM, which includes detailed descriptions of all food and amounts consumed by subjects. As per WWEIA protocols, all participants were eligible for two 24 h dietary recall interviews. The first dietary recall interview was collected in-person in the Mobile Examination Center (MEC), and the second interview was collected by telephone 3 to 10 days later. Parents or caregivers provided dietary intake information. While two days of 24 h dietary recalls were collected in WWEIA, the current analysis used Day 1 data as this represents the in-person data collection in the MEC [18,19].

### 2.2. Methods and Statistical Analysis

All statistical analyzes were performed using SAS software (Version 9.2, SAS Institute, Cary, NC, USA) and SUDAAN 11.0. Survey weights were used to generate nationally-representative estimates for infants, which were also adjusted for the complex sample design of NHANES. Adjusted least-square means and standard errors of daily energy, nutrient intakes and diet quality were determined. Energy intake and diet quality components were adjusted for age, gender, ethnicity, and poverty income ratio (PIR). Nutrients, body weight, and recumbent length were adjusted for the same covariates, but also for energy intake. Diet quality was assessed using USDA’s Healthy Eating Index 2010 (HEI)-2010 and considered both the total and sub-component scores. The HEI-2010 provides a measure of diet quality and measures conformance to federal dietary guidance and has been predominantly used to monitor dietary practices of the US population and the low-income subpopulation [22]. While the HEI has been developed for those ≥2 years of age and given that most HEI measures are adjusted for energy (kilocalories), the HEI tool was considered valid for use in infants in the present analysis.

Socioeconomic status was assessed using the PIR, which was grouped into three categories (<1.35, 1.35 ≤ PIR ≤ 1.85, and >1.85), reflecting the federally established poverty criteria, thus a PIR of <1.35 equated to below 135% of poverty, while higher PIR values represented the subject was from a higher income family. Additional analyzes were completed considering food security status (low food security vs. high food security) and whether the mother of the infants participated in the Special Supplemental Nutrition Program for Women, Infants, and Children (WIC). WIC provides federal grants to the various US states for supplemental foods, health care referrals, and nutrition education for low-income pregnant, breastfeeding, and non-breastfeeding postpartum women, and to infants and children up to the age of five who are found to be at nutritional risk [23].

## 3. Results

### 3.1. Study Participants

Demographics of the infant study population are presented in Table 1. Comparisons between egg consumers and non-consumers showed significant differences in age, PIR < 1.35, PIR > 1.85 (i.e., a higher PIR indicates a higher income) and WIC status, with no differences seen in the other variables.

### 3.2. Energy and Nutrient Intakes in Infants

Adjusted energy and nutrient intake data for egg consumers and egg non-consumers are presented in Table 2. Consumption of eggs in infants was associated with greater intake of several nutrients, compared to infants not consuming eggs. This included significantly higher intake of protein, lutein + zeaxanthin, choline, B12, octadecatrienoic acid (i.e., α-linolenic acid), DHA, selenium and phosphorus; and lower added and total sugar. Relative to egg non-consumers, egg consumers also had less of several nutrients, which should be encouraged, including significantly lower daily intakes of vitamin A, D, E, dietary fiber, iron and potassium. Egg consumers also had a significantly higher daily intake of saturated fat and sodium.

### 3.3. Diet Quality: USDA Healthy Eating Index-2010 Scores in Infants

Mean total and sub-component HEI scores are presented in Table 3 for egg consumers and egg non-consumers. No significant differences were seen between egg consumers and non-consumers for HEI total score. While egg consumption is not associated with overall diet quality in infants, egg consumers had higher scores for several HEI sub-components. Specifically, egg consumers had greater scores for green beans, dairy, total protein foods and lower scores for sodium. Egg consumers also had lower scores for whole fruit in comparison to egg non-consumers.

### 3.4. Added Sugar, Lutein + Zeaxanthin Intake and Socioeconomic Status in Infants

An additional analysis was completed that examined added sugar and lutein + zeaxanthin intake by PIR, WIC and food security status (see Table 4). Egg consumption in infants 6–24 months of age, regardless of socioeconomic status, as assessed by PIR, WIC, and food security status, is associated with greater lutein + zeaxanthin and lower added sugar intake per day in comparison to egg non-consumers. Furthermore, regardless of socioeconomic status, DHA intake was significantly higher in egg consumers relative to egg non-consumers.

### 3.5. Weight and Recumbent Length in Infants

No significant differences were seen in weight between egg consumers and egg non-consumers. Recumbent length was significantly greater in infants consuming eggs relative to infants not including eggs in their diet (see Table 5).

## 4. Discussion

To our knowledge, this is the first NHANES study examining egg consumption and links to nutrient intakes and growth-related outcomes in an infant population. Consumption of eggs in 6 to 24 month-olds was associated with higher nutrient intakes compared to infant egg non-consumers. Specifically, egg consumption was associated with significantly higher intake of protein, lutein + zeaxanthin, choline, B12, selenium and phosphorus, and lower intake of added and total sugars per day relative to non-consumers of eggs. Nonetheless, infant egg consumers also had lower intakes of vitamin A, D, E, dietary fiber, iron and potassium vs. egg non-consumers, suggesting the complementary foods, including baby cereals, fruits, vegetables, may be required to enhance the infant dietary pattern. Infant egg consumers also had a greater intake of saturated fat and sodium; thus, preparation and delivery of eggs needs further assessment. While egg consumption was not associated with overall diet quality, egg consumers had higher scores for several Healthy Eating Index 2010 sub-components, including greater scores for green beans, dairy, total protein foods and lower scores for sodium. However, egg consumers also had lower scores for whole fruit vs. egg non-consumers, suggesting that caregivers may need to be more attentive to adding additional fruit to infant diets. Infant egg consumers had greater intake of protein, lutein + zeaxanthin, choline, vitamin B12, selenium and phosphorus, however, egg consumers also had lower intake of several nutrients to be encouraged in the diet (i.e., dietary fiber/iron) and greater intake of nutrients to limit (i.e., sodium/saturated fat). Therefore, dietary strategies that target increased consumption of nutrient-dense foods lower in sodium and saturated fat, in combination with eggs, may prove nutritionally valuable in US infants aged 6 to 24 months.

The current analysis has shown that egg consumers have a greater daily intake of octadecatrienoic acid (i.e., α-linolenic acid and DHA) in comparison to egg non-consumers which aligns with published studies. Specifically, researchers previously examined the impact of consuming one egg per day for six months on choline, vitamin B12 and essential fatty acid status [13]. The eggs used in the randomized controlled trial provided >50% of the daily infant (ages 7–12 months) Recommended Dietary Allowance requirements for protein, vitamin B12 and nearly 96% of the Adequate Intake for choline. The researchers measured three essential fatty acids (linoleic acid, α-linolenic acid, and DHA). The egg intervention significantly increased DHA plasma concentrations relative to the control, with a notable effect size of 0.43. Infancy, childhood and adolescence are documented as developmental periods with neuronal maturation, synaptogenesis and gray matter expansion, all of which are linked with DHA accumulation [24,25]. However, DHA synthesis from its fatty acid precursors is inefficient, thus establishing the need for dietary sources of DHA. This is coupled with few foods naturally providing appropriate levels of DHA [6,9]. As DHA has been shown to be a fundamental fatty acid in growth, neurodevelopment and function, DHA concurrently may be lacking in the diet of young children worldwide [11]. Thus, egg consumption can represent an important component of infant and toddler dietary patterns. Further, in our current analysis, we observed that recumbent length was significantly greater in infant egg consumers vs. egg non-consumers, with no differences seen when evaluating body weight. While infant growth and development is multifactorial, further research on infants, children and adolescents are required to assess the importance of eggs in dietary patterns throughout the various stages of infancy and childhood.

In additional analyzes, egg consumption, regardless of socioeconomic status, was associated with greater lutein + zeaxanthin and lower added sugar intake per day compared to non-consumers of eggs, thus, further emphasizing the nutritional benefits linked to eggs. Previous nutritional economic research using data from the USDA Center for Nutrition Policy and Promotion food price database [26] found that eggs were the lowest cost source for protein, vitamin A, iron, vitamin B12, riboflavin, and the second lowest cost source for zinc and calcium [27].

Introduction of protein-rich foods may be delayed by caregivers worldwide due to allergy and food sensitivity-related anxieties. The 2010 United Kingdom Infant Feeding Survey reported 45% of mothers of infants 8 to 10 months of age avoided giving their infant eggs, nuts, dairy and/or fish, with fear of allergy being documented as the rationale for egg avoidance and/or delay in egg introduction, followed by perception that the infant was too young [28]. The American Academy of Pediatrics (AAP) Committee on Nutrition and Section on Allergy and Immunology conducted a review to provide guidance to clinicians in pediatric care. The committee examined the nutritional options during pregnancy, lactation, and the first year of life that may affect the development of atopic disease, including atopic dermatitis, asthma and food allergy [4]. When considering breastfeeding practices, past AAP positioning has recommended lactating mothers with infants at high risk of developing an allergy to avoid peanuts and tree nuts, and consider the elimination of eggs, milk and fish while breastfeeding, since previous studies have demonstrated benefits linked to this dietary practice [2]. Similarly, AAP has also recommended solid foods be delayed until 4 to 6 months of age, with whole cow’s milk being introduced at 12 months [4]. Previous AAP recommendations for infant diets also included the avoidance of eggs until two-years of age for infants identified at risk of developing atopic disease [2]. Current AAP position statements establish a lack of substantiation that maternal dietary restrictions during pregnancy and/or lactation prevent atopic disease, and while recommendations maintain that solid foods should not be introduced before 4 to 6 months of age. AAP has stated there is no convincing evidence that delaying eggs, fish and peanut-containing foods beyond this period has a protective effect on the development of atopic disease [4]. Thus, some health professionals suggest that the introduction of eggs during infancy does not increase allergy incidence risk or egg sensitivity, as has been previously communicated by authoritative sources. This may have led to parents/caregivers avoiding and/or delaying egg and egg-containing meal introduction to infants and toddlers.

The current analyzes have limitations inherent in observational research and have previously been reported in similar research designs. The results are dependent on self-reported dietary data for foods, which may involve study participants under- or over-estimating food consumption, leading to inaccuracies in energy and nutrient intakes. Data were also obtained using a 24 h dietary recall, which relies on the study caregiver’s memory, and while validated methods are used to gather the data, recall information is subject to inaccuracies and bias from memory challenges and other potential measurement errors experienced in epidemiological investigations [29]. It is also important to recognize that the current study considered dietary patterns with and without egg consumption, hence, other foods within the dietary patterns, may also contribute to the associations observed with nutrient intakes. A significant benefit of using NHANES data for the current analyzes includes access to a large and nationally-representative dataset of adults of various age groups in the US and corresponding food and nutrient intake data. As the present research is observational, and since growth and development are multifactorial, future research designs will need to consider randomized controlled trials.

## 5. Conclusions

This is the first study in US infants to demonstrate differences in nutrient intakes between egg consumers and non-consumers. Egg consumption in US infants was linked to higher consumption of several key nutrients important for growth and development, lower intakes of added and total sugars and higher recumbent length. Introducing eggs into infant dietary patterns early (i.e., six months of age) may help contribute to optimal nutrient intakes, particularly when considering protein, choline, vitamin B12, lutein + zeaxanthin, α-linolenic acid, DHA, and phosphorus, all of which play imperative functions in growth and development. Egg consumers also had less of several nutrients to be encouraged and greater intake of nutrients to limit in the diet, thus presenting opportunities for educational strategies that focus on increased consumption of nutrient-dense foods (i.e., grains, fruits, vegetables, and dairy) that are lower in sodium and saturated fats. Caregivers may consider dietary patterns that also include selections from the grains, fruits/vegetables, and dairy food groups, in combination with eggs. In contrast, avoiding and/or delaying egg consumption may exacerbate nutrient shortfalls in infants. Lastly, as infant egg consumers also had lower intakes of several nutrients to be encouraged, concurrently with greater intake of nutrients to limit, future nutritional and educational strategies may consider promoting dietary patterns that increase consumption of lower sodium, nutrient-dense foods in combination with the early introduction of eggs.

## Figures and Tables

**Table 1:** Mean demographic variables in egg consumers compared to egg non-consumers.

Variable	Egg Non-Consumers		Egg Consumers		*p*
	LSMean	SE	LSMean	SE	
Age (Months)	14.60	0.14	16.00	0.23	<0.0001
Gender, Male (%)	48.54	1.42	52.80	2.74	0.1679
PIR < 1.35 (%)	36.53	1.37	51.69	2.97	<0.0001
1.35 ≤ PIR ≤ 1.85 (%)	11.72	1.00	10.17	1.78	0.4484
PIR > 1.85 (%)	51.75	1.69	38.13	3.29	0.0002
WIC Participant (%)	47.10	1.69	55.45	3.85	0.0472
Full Food Security (%)	69.65	1.38	63.97	2.98	0.0841

LSMean = least square mean; SE = standard error; PIR = poverty income ratio; WIC = Special Supplemental Nutrition Program for Women, Infants and Children; Data represent infants 6–24 months-old; Data source; NHANES 2001–2012 using Day 1 intakes.

**Table 2:** Adjusted daily energy and nutrient intakes in egg consumers vs. egg non-consumers using NHANES 2001–2012; Infants 6–24 months-old.

Energy/Nutrients	Egg Non-Consumers		Egg Consumers		Beta	SE	*p*
	LSMean	SE	LSMean	SE			
Energy (kcal)	1190	14	1265	27	75	28	0.0103
Carbohydrate (g)	163.0	0.8	142.4	1.8	−20.6	2.1	<0.0001
Added sugars (tsp eq)	6.1	0.2	4.7	0.3	−1.3	0.4	0.0006
Total sugars (g)	98.5	0.8	87.3	1.8	−11.2	2.1	<0.0001
Protein (g)	41.3	0.4	48.0	0.7	6.7	0.8	<0.0001
Total fat (g)	44.6	0.3	50.5	0.6	5.9	0.7	<0.0001
Total monounsaturated fatty acids (g)	15.0	0.1	17.2	0.2	2.2	0.3	<0.0001
Total polyunsaturated fatty acids (PFA) (g)	7.8	0.1	8.2	0.2	0.4	0.3	0.0806
Total saturated fatty acids (g)	18.1	0.2	20.5	0.3	2.3	0.4	<0.0001
PFA 18:3 (Octadecatrienoic) (g)	0.820	0.008	0.872	0.019	0.052	0.023	0.0282
PFA 20:5 (Eicosapentaenoic) (g)	0.005	0.001	0.006	0.001	0.001	0.001	0.2373
PFA 22:6 (Docosahexaenoic) (g)	0.022	0.001	0.042	0.002	0.020	0.002	<0.0001
Cholesterol (mg)	97.0	1.3	328.6	8.5	231.6	8.7	<0.0001
Dietary fiber (g)	7.7	0.1	6.9	0.2	−0.8	0.2	0.0004
Calcium (mg)	970.0	11.2	953.9	24.1	−16.1	28.2	0.5694
Folate, DFE (mcg)	301.4	4.7	271.4	10.3	−30.0	11.9	0.0135
Iron (mg)	12.6	0.2	10.0	0.3	−2.6	0.4	<0.0001
Lutein + zeaxanthin (mcg)	532.5	23.2	787.8	63.8	255.3	67.1	0.0003
Magnesium (mg)	165.2	1.2	159.8	2.0	−5.4	2.4	0.0293
Niacin (mg)	12.8	0.2	10.5	0.3	−2.3	0.3	<0.0001
Phosphorus (mg)	902.5	7.8	977.3	15.0	74.8	18.4	0.0001
Potassium (mg)	1802.1	12.6	1839.7	29.7	37.6	34.5	0.2789
Riboflavin (Vitamin B2) (mg)	1.81	0.02	1.88	0.03	0.07	0.03	0.0536
Selenium (mcg)	51.8	0.6	66.7	1.0	14.9	1.1	<0.0001
Sodium (mg)	1418.1	19.1	1663.0	36.0	244.9	42.4	<0.0001
Thiamin (Vitamin B1) (mg)	1.12	0.01	0.96	0.02	−0.16	0.02	<0.0001
Total choline (mg)	162.7	1.6	281.1	5.9	118.3	6.2	<0.0001
Vitamin A, RAE (mcg)	596.6	10.1	605.6	21.2	9.1	23.5	0.6998
Vitamin B12 (mcg)	3.7	0.1	4.2	0.1	0.5	0.2	0.0026
Vitamin B6 (mg)	1.10	0.01	1.06	0.03	−0.04	0.03	0.1765
Vitamin C (mg)	93.6	2.5	86.2	4.0	−7.4	5.0	0.1396
Vitamin D (D2 + D3) (mcg)	8.5	0.1	9.0	0.3	0.5	0.3	0.1038
Vitamin E as alpha-tocopherol (mg)	5.1	0.1	4.9	0.2	−0.1	0.2	0.4521
Zinc (mg)	7.3	0.1	7.3	0.1	0.0	0.1	0.8897

LSMean = least square mean; SE = standard error; Beta = regression coefficient for difference among groups; Data represent egg consumers (*N* = 561) and egg non-consumers (*N* = 2129); infants 6–24 months-old, gender combined; NHANES 2001–2012; Day 1 intakes; Covariates include age, gender, ethnicity, poverty income ratio, and for all variables except ‘Energy’, the covariate of energy (kcal).

**Table 3:** Adjusted mean (SE) Total Healthy Eating Index-2010 (HEI) and Sub-Component Scores for egg consumers vs. egg non-consumers using NHANES 2001–2012; 6–24 months old.

HEI Total and Sub-Component Scores	Egg Non-Consumers		Egg Consumers		SE	*p*
	LSMean	SE	LSMean	SE		
HEI-2010 component 1 (total vegetables)	1.91	0.04	1.95	0.10	0.09	0.6942
HEI-2010 component 2 (greens and beans)	0.40	0.04	0.64	0.09	0.09	0.0085
HEI-2010 component 3 (total fruit)	3.76	0.06	3.85	0.10	0.12	0.4697
HEI-2010 component 4 (whole fruit)	3.28	0.06	2.90	0.12	0.14	0.0073
HEI-2010 component 5 (whole grains)	2.47	0.09	1.80	0.14	0.17	0.0002
HEI-2010 component 6 (dairy)	6.85	0.10	7.60	0.17	0.20	0.0003
HEI-2010 component 7 (total protein foods)	2.19	0.05	3.68	0.08	0.10	<0.0001
HEI-2010 component 8 (seafood and plant protein)	0.88	0.05	0.91	0.10	0.11	0.8345
HEI-2010 component 9 (fatty acid ratio)	1.85	0.07	1.58	0.13	0.16	0.0857
HEI-2010 component 10 (sodium)	7.75	0.09	6.57	0.22	0.25	<0.0001
HEI-2010 component 11 (refined grains)	8.13	0.10	8.56	0.18	0.21	0.0427
HEI-2010 component 12 (sofaas calories)	15.32	0.15	14.75	0.31	0.34	0.0985
**HEI-2010 TOTAL SCORE**	54.80	0.29	54.78	0.59	0.64	0.9753

LSMean = least square mean; SE = standard error; Beta = regression coefficient for difference among groups; Data represent egg consumers (*N* = 561) and non-consumers (*N* = 2129); infants aged 6–24 months-old, gender combined; NHANES 2001–2012; Day 1 intakes; Covariates include age, gender, ethnicity, and poverty income ratio.

**Table 4:** Adjusted mean (SE), added sugar, lutein + zeaxanthin, octadecatrienoic acid, eicosapentaenoic acid, and docosahexaenoic acid based on socioeconomic measures for egg consumers vs. egg non-consumers.

Population	Nutrient	Egg Non-Consumers			Egg Consumers			*p*
		*N*	LSMean	SE	*N*	LSMean	SE	
Food Security = High	Added sugars (tsp eq)	1278	5.9	0.2	309	4.9	0.5	0.0709
Food Security = High	Lutein + zeaxanthin (mcg)	1278	555.4	31.6	309	797.0	83.4	0.0046
Food Security = High	PFA 18:3 (Octadecatrienoic) (g)	1278	0.816	0.011	309	0.841	0.024	0.3980
Food Security = High	PFA 20:5 (Eicosapentaenoic) (g)	1278	0.005	0.001	309	0.006	0.001	0.2883
Food Security = High	PFA 22:6 (Docosahexaenoic) (g)	1278	0.021	0.001	309	0.042	0.003	<0.0001
Food Security = Low	Added sugars (tsp eq)	812	6.4	0.3	236	4.3	0.4	<0.0001
Food Security = Low	Lutein + zeaxanthin (mcg)	812	487.8	44.8	236	721.1	83.9	0.0383
Food Security = Low	PFA 18:3 (Octadecatrienoic) (g)	812	0.827	0.016	236	0.922	0.026	0.0036
Food Security = Low	PFA 20:5 (Eicosapentaenoic) (g)	812	0.004	0.001	236	0.005	0.001	0.6578
Food Security = Low	PFA 22:6 (Docosahexaenoic) (g)	812	0.026	0.002	236	0.042	0.003	0.0001
PIR < 1.35	Added sugars (tsp eq)	1058	6.8	0.3	325	6.1	0.5	0.2509
PIR < 1.35	Lutein + zeaxanthin (mcg)	1058	515.6	42.5	325	786.8	92.2	0.0065
PIR < 1.35	PFA 18:3 (Octadecatrienoic) (g)	1058	0.844	0.016	325	0.906	0.023	0.0554
PIR < 1.35	PFA 20:5 (Eicosapentaenoic) (g)	1058	0.005	0.001	325	0.006	0.001	0.4356
PIR < 1.35	PFA 22:6 (Docosahexaenoic) (g)	1058	0.024	0.001	325	0.039	0.003	<0.0001
PIR ≥ 1.35	Added sugars (tsp eq)	952	5.5	0.2	203	3.6	0.3	<0.0001
PIR ≥ 1.35	Lutein + zeaxanthin (mcg)	952	544.1	25.7	203	783.0	80.7	0.0034
PIR ≥ 1.35	PFA 18:3 (Octadecatrienoic) (g)	952	0.804	0.011	203	0.838	0.024	0.2187
PIR ≥ 1.35	PFA 20:5 (Eicosapentaenoic) (g)	952	0.005	0.001	203	0.006	0.001	0.2521
PIR ≥ 1.35	PFA 22:6 (Docosahexaenoic) (g)	952	0.021	0.002	203	0.045	0.003	<0.0001
WIC Participant = No	Added sugars (tsp eq)	650	5.7	0.2	139	4.1	0.7	0.0260
WIC Participant = No	Lutein + zeaxanthin (mcg)	650	546.0	29.2	139	853.0	109.1	0.0040
WIC Participant = No	PFA 18:3 (Octadecatrienoic) (g)	804	0.816	0.011	180	0.866	0.032	0.1469
WIC Participant = No	PFA 20:5 (Eicosapentaenoic) (g)	804	0.004	0.001	180	0.008	0.001	0.0200
WIC Participant = No	PFA 22:6 (Docosahexaenoic) (g)	804	0.020	0.002	180	0.045	0.004	<0.0001
WIC Participant = Yes	Added sugars (tsp eq)	1082	5.9	0.2	304	5.3	0.4	0.1143
WIC Participant = Yes	Lutein + zeaxanthin (mcg)	1082	493.6	42.4	304	783.1	101.9	0.0123
WIC Participant = Yes	PFA 18:3 (Octadecatrienoic) (g)	1319	0.823	0.015	375	0.881	0.022	0.0481
WIC Participant = Yes	PFA 20:5 (Eicosapentaenoic) (g)	1319	0.005	0.001	375	0.005	0.001	0.7046
WIC Participant = Yes	PFA 22:6 (Docosahexaenoic) (g)	1319	0.025	0.002	375	0.040	0.003	<0.0001

LSMean = least square mean; SE = standard error; Data were gender combined for infants 6–24 months-old; NHANES 2001–2012; PFA = polyunsaturated fatty acid; PIR = poverty income ratio; WIC = Special Supplemental Nutrition Program for Women, Infants, and Children; Day 1 intakes; Covariates include age, gender, ethnicity, energy (kcal), and poverty income ratio (except when examining the PIR population).

**Table 5:** Adjusted mean (SE) recumbent length for egg consumers vs. egg non-consumers.

Growth Variable	Egg Non-Consumers			Egg Consumers			*p*
	*N*	LSMean	SE	*N*	LSMean	SE	
Weight (kg)	2040	10.7	0.04	533	10.9	0.1	0.1497
Recumbent Length (cm)	2105	78.7	0.1	550	79.2	0.2	0.0298

LSMean = least square mean; SE = standard error; Data were gender combined; NHANES 2001–2012; Day 1 intakes; Covariates include age, gender, ethnicity, poverty income ratio, and energy intake (kcal).
